# Effects of physical exercise on cognitive impairment in patients with Alzheimer’s disease, Parkinson’s disease or mild cognitive impairment: a systematic review and meta-analysis

**DOI:** 10.1186/s11556-026-00412-2

**Published:** 2026-04-22

**Authors:** Iris Kruijff, Erwin E.H. van Wegen, Joram D. Mul, Richard T. Jaspers, Anouk Schrantee, Erik J.A. Scherder, Paul J. Lucassen, Anne-Marie van Dam

**Affiliations:** 1 Amsterdam UMC, Vrije Universiteit Amsterdam, Dept. Anatomy and Neurosciences, Amsterdam, The Netherlands; 2 Vrije Universiteit Amsterdam, Center for Neurogenomics and Cognitive Research, Dept. Functional Genomics, Amsterdam, The Netherlands; 3 Amsterdam UMC, Vrije Universiteit Amsterdam, Alzheimer Centre, Dept. Neurology, Amsterdam, Netherlands; 4 Amsterdam Neuroscience, Amsterdam, The Netherlands; 5 Amsterdam UMC, Vrije Universiteit Amsterdam, Dept. Rehabilitation Medicine, Amsterdam, The Netherlands; 6 Amsterdam Movement Sciences, Amsterdam, The Netherlands; 7 University of Amsterdam, Faculty of Science, Swammerdam Institute for Life Sciences, Brain Plasticity group, Amsterdam, The Netherlands; 8 University of Amsterdam, Centre for Urban Mental Health, Amsterdam, The Netherlands; 9 Vrije Universiteit Amsterdam, Faculty of Behavioural and Movement Sciences, Dept. Human Movement Sciences, Laboratory for Myology, Amsterdam, The Netherlands; 10 Amsterdam UMC, University of Amsterdam, Dept. Radiology and Nuclear Medicine, Amsterdam, The Netherlands; 11 Vrije Universiteit Amsterdam, Dept. Clinical Neuropsychology, Amsterdam, The Netherlands

**Keywords:** Alzheimer’s disease, Mild cognitive impairment, Parkinson’s disease, Neurodegenerative disorders, Physical exercise, Global cognition, Executive function, Memory, Attention

## Abstract

**Background:**

Neurodegenerative disorders impose a major burden on patients and their families worldwide, and finding effective ways to treat or prevent these disorders is essential but challenging. In recent years, physical exercise interventions have attracted considerable attention as a potential approach to modify cognitive decline, which is a major shared symptom of these disorders. In this systematic review and meta-analysis, we focused on studies investigating the effectiveness of physical exercise interventions for various cognitive domains in patients with Alzheimer’s disease (AD), Parkinson’s disease (PD) or mild cognitive impairment (MCI), who presented with initial cognitive deficits.

**Methods:**

We performed a systematic literature search in PubMed, Embase and CENTRAL for randomized controlled trials (RCTs) published in English until October 2025. We considered them eligible when they investigated the effects of physical exercise on cognitive functioning in AD, PD or MCI patients who all already had cognitive deficits before the start of the study. A random-effects model was applied to explore the effects of physical exercise on four main cognitive domains: global cognition, executive function, memory and attention.

**Results:**

In total, 28 RCTs were eligible for inclusion in this review (AD *n* = 8; MCI *n* = 16; PD *n* = 4). Patient subgroup analyses revealed that physical exercise significantly improved global cognition, executive function, memory and attention in both the MCI and AD patient groups, whereas no significant effects were observed in PD patients.

**Conclusions:**

These findings indicate that exercise-related benefits extend across multiple cognitive domains in AD and MCI patients and support its potential as a non-pharmacological strategy for individuals with neurodegenerative disorders. In PD, evidence remains limited and inconclusive.

Despite substantial variability in exercise interventions, consistent effects were observed, suggesting that cognitive benefits may be broadly applicable across different modalities. However, the limited number of studies including patients with objectively verified cognitive impairment, particularly in PD, as well as the focus on global cognition in many AD studies, highlight important gaps in the current literature. Future research should specifically target cognitively impaired populations and include sufficiently large and well-characterized samples to improve the precision and clinical applicability of the evidence.

## Introduction

Neurodegenerative disorders have a major impact on the quality of life of many patients, their relatives and caretakers, and as such, on global health care [1]. Although the aetiology and neuropathology of these disorders are diverse, cognitive impairment is a common symptom among various neurodegenerative diseases, including Alzheimer’s disease (AD) and Parkinson’s disease (PD) [2, 3].

Cognition involves general functions such as perception, learning and memory, consciousness, and language, and impaired cognition can affect multiple domains, including memory, attention, executive function, processing speed, visuospatial function and social cognition, which together strongly influence an individual’s functioning and independence. Cognitive decline ranges from mild forms, such as a slow and initially mild decline in normal aging and mild cognitive impairment (MCI) [4], to more moderate and severe, incapacitating forms occurring during neurodegenerative disorders, such as AD and PD [5].

MCI patients, although still capable of performing daily activities, exhibit cognitive deficits that are more prominent than expected for their age, and is therefore seen by many as a prodromal stage before dementia ensues [5]. In patients with AD, the most common cause of dementia, a gradual decline in learning and memory occurs, which is then followed by further worsening and impairments in executive functions and other cognitive domains, resulting in complete dependence on care in the latest stages of disease [2]. PD patients primarily suffer from motor dysfunctions, such as bradykinesia and tremor, alongside significant cognitive decline [6]. Approximately 2–50% of PD patients develop MCI [3, 7], and approximately 80% eventually develop Parkinson’s disease dementia (PDD) [8]. Cognitive impairments in PD patients can be heterogeneous but primarily affect executive and visuospatial functions, attention and processing speed [7, 9].

These cognitive impairments severely impact daily functioning and quality of life for patients, yet effective treatments to prevent or delay cognitive decline are currently not available. In recent years, there has been a growing interest in behavioural interventions, such as physical exercise training, with the aim of stabilizing or possibly even reversing the cognitive decline associated with these disorders. Beneficial effects of exercise on cognition are thought to be mediated by multiple, interrelated mechanisms at molecular, cellular and systemic levels. Physical activity upregulates neurotrophic factors such as brain-derived neurotrophic factor (BDNF), insulin-like growth factor (IGF-1) and vascular endothelial growth factor (VEGF), that have been linked to hippocampal neurogenesis, synaptogenesis and enhanced synaptic plasticity [10–14]. In addition, exercise improves cerebral blood flow and metabolic efficiency, thereby enhancing oxygen and glucose delivery to the brain [15]. Anti-inflammatory and antioxidant effects of regular physical activity further contribute to neuroprotection by reducing oxidative stress and dampening chronic neuroinflammation, processes that are strongly implicated in neurodegenerative pathology [16, 17].

Preclinical studies in relevant mouse models have shown positive effects of physical exercise on various measures of cognition. For example, treadmill running in a triple-transgenic mouse model of AD improved cognitive performance in the Morris water maze [18], a paradigm used to test spatial memory. In addition, in a DJ-1 gene knockout mouse model of PD, voluntary wheel running improved cognition [19].

Individual clinical studies have also demonstrated positive effects of physical exercise on cognition in neurological patients. For example, thirty minutes of treadmill walking twice a week for three months improved global cognition in patients with AD [20]. Similarly, 16 weeks of aerobic training, twice a week with an increasing duration of 20–45 min per session, improved executive function in PD patients [21]. However, not all studies reported positive findings, e.g. eight weeks of thrice-weekly one-hour aerobic interval training failed to improve cognitive function in patients with PD [22]. Additionally, moderate- to high-intensity physical exercise training for 16 weeks does not seem to improve cognition in AD patients [23].

It is important to note that patients with neurodegenerative disorders experience impairments across multiple cognitive domains. During disease progression, patients with AD, PD or MCI all suffer from impairments in global cognition, executive function, memory and attention. Thus, synthesizing results at the level of multiple relevant cognitive domains is important to understand which aspects of cognition may benefit from physical exercise. In the present systematic review and meta-analysis of randomized controlled trials (RCTs), we therefore aim to analyse the effects of physical exercise interventions versus no-intervention on the above mentioned four key domains, i.e. global cognition, executive function, memory, and attention in patients with AD, PD or MCI. By focusing only on patients who already experience objective cognitive deficits before intervention starts, our analysis reflects a clinically relevant disease stage at which exercise may help prevent further cognitive decline or improve functioning. This may provide insight into the cognitive domain-specific effects of exercise across patients with AD, PD or MCI, which can be a starting point for future research on physical exercise intervention strategies on patients with cognitive impairment.

## Materials and methods

### Literature search strategy

The databases PubMed, Embase and Cochrane Central Register of Controlled Trials (CENTRAL) were searched for RCTs published until October 2025. In PubMed, article type was filtered for ‘Randomized Controlled Trial’ and the following search string was used to find applicable articles: (“Alzheimer Disease“[Mesh] OR “Parkinson Disease“[Mesh] OR “Mild Cognitive Impairment“[Mesh] OR Alzheimer* OR Parkinson* OR “mild cognitive impairment” OR MCI) AND (“Exercise“[Mesh] OR “Motor Activity“[Mesh] OR “Physical Fitness“[Mesh] OR “Exercise Therapy“[Mesh] OR exercise OR “physical activity” OR “physical exercise” OR training OR “exercise training” OR “aerobic training” OR “resistance training”) AND (“Cognition“[Mesh] OR “Cognitive Function“[Mesh] OR “Executive Function“[Mesh] OR “Memory“[Mesh] OR “Attention“[Mesh] OR cognition OR “cognitive function” OR “executive function” OR “global cognition” OR memory OR attention). For Embase, article type was also filtered for ‘Randomized Controlled Trial’ and the following search string was applied: (‘Alzheimer disease’/exp OR’ Parkinson disease’/exp OR ‘mild cognitive impairment’/exp OR Alzheimer* OR Parkinson* OR “mild cognitive impairment” OR MCI) AND (‘exercise’/exp OR ‘motor activity’/exp OR ‘physical fitness’/exp OR ‘exercise therapy’/exp OR ‘aerobic exercise’/exp OR ‘resistance training’/exp OR exercise OR “physical activity” OR “physical exercise” OR training OR “exercise training” OR “aerobic training” OR “resistance training”) AND ( ‘cognition’/exp OR ‘cognitive function’/exp OR ‘executive function’/exp OR ‘memory’/exp OR ‘attention’/exp OR cognition OR “cognitive function” OR “executive function” OR “global cognition” OR memory OR attention). An additional search in CENTRAL was performed, selecting only for papers that were not found in PubMed or Embase, by using the following search string: (“Alzheimer” OR “Parkinson” OR “mild cognitive impairment”) AND (“exercise” OR “physical activity” OR “physical exercise” OR “training” OR “exercise training” OR “aerobic training” OR “resistance training”) AND (“cognition” OR “cognitive function” OR “executive function” OR “global cognition” OR “memory” OR “attention”).

### Eligibility criteria

Studies were considered eligible if they met the following *inclusion criteria*:

1. Study participants were diagnosed with AD, PD or MCI.
2. Participants presented with objective cognitive impairments at baseline, as determined by a global cognition test (i.e., MMSE < 24 or MoCA < 26).
3. The intervention group received a physical exercise intervention not combined with other cognition-affecting interventions.
4. The control group received usual care without any physical exercise or cognition-enhancing intervention. Adjustments for differences in social attention between the control and intervention group were allowed.
5. At least one cognitive outcome measure was reported.
6. The study was a randomized controlled trial (RCT).

The* exclusion criteria* were as follows:

1. The study was a preclinical study, case study, review, systematic review, meta-analysis, or protocol.

### Study selection and data extraction

Study selection and data extraction were performed by one primary reviewer (first author). Search hits were first selected for eligibility based on their title. The abstracts and subsequent relevant full texts were screened for the inclusion and exclusion criteria, and only eligible articles were included in the meta-analysis. When there was doubt, a secondary reviewer (last author) was consulted to achieve consensus. The following data were extracted: author, neurological disease of the study participants, average age of the participants in each group, initial cognitive status, type of exercise intervention, frequency and duration of intervention and cognitive outcome measures.

In addition, from all the articles appropriate for the meta-analysis, the means and standard deviations of all the cognitive measures before and after the intervention, and the number of participants in the control and intervention groups were extracted to determine the standardized mean difference (SMD) and confidence interval (CI). Information from four RCTs eligible for the meta-analysis was lacking. The corresponding authors were contacted, of which one provided the missing data on request, the other three did not and thus these RCTs were excluded from the analysis.

### Categorization of tests in cognitive domains

In this study, the cognitive tests were grouped into four primary cognitive domains – global cognition, executive function, memory and attention – based on classical neuropsychological frameworks [24]. Global cognition refers to overall cognitive functioning and general mental status. Measures in this domain provide a broad assessment of cognitive abilities across multiple domains [24]. Executive function refers to higher-order cognitive processes involved in planning, cognitive flexibility, inhibition, manipulation of information, and strategic retrieval. Tasks in this domain require participants to actively manipulate information or control automatic responses [24]. Memory encompasses the encoding, storage, and retrieval of information. Tasks in this domain assess both short- and long-term memory [24]. Attention refers to the ability to sustain focus, rapidly process information, and selectively attend to relevant stimuli. Tasks in this domain primarily measure processing speed and visual scanning without substantial manipulation or inhibitory demands [24]. We acknowledge the fact that neuropsychological tests often cover multiple cognitive domains, and we therefore classified them based on the domain they primarily measure.

### Assessment of risk of bias in selected articles

All studies eligible for the meta-analysis were assessed for their risk of bias via the Cochrane risk-of-bias tool for randomized controlled trials (RoB 2) [25]. This tool evaluates five domains of bias: [1] bias due to randomization [2], bias due to deviations from intended intervention [3], bias due to missing data [4], bias due to outcome measurement, and [5] bias due to selection of reported result. Each domain was judged as ‘low risk of bias’, ‘some concerns’, or ‘high risk of bias’, in accordance with the RoB 2 guidance. An overall risk-of-bias judgement was then assigned for each study based on these domain-level assessments. Studies were classified as having an overall low risk of bias if all domains were rated as low risk. If at least one domain raised some concerns and no domains were rated as high risk, the overall judgement was ‘some concerns’. An overall high risk of bias was assigned if at least one domain was judged as high risk of bias, in line with Cochrane recommendations [26].

### Data analysis

Data analysis was performed on the cognitive outcome measures of the RCTs. The metafor package in R was used to compute the effect size (ES) per outcome measure based on the change in cognitive outcome scores caused by the exercise intervention, i.e. the change score (post-intervention mean score minus pre-intervention mean score), divided by the pooled standard deviation, resulting in the standardized mean difference (SMD). Scores of cognitive tests that show higher scores for greater impairment were reversed, so that positive SMDs consistently reflected improvement in cognitive performance. When a RCT reported more than one outcome measure within the same cognitive domain, we combined these outcomes into a single ES using a weighted average of the SMDs. Weights among the different outcome measures were determined based on the variance, allowing outcomes with greater precision to contribute more strongly to the combined estimate [27]. Five studies used two different exercise interventions, but with a shared control group [15, 28–31]. We included both exercise interventions in our analysis, but equally divided the sample size of the control group over the two experimental groups to ensure appropriate contribution to the analysis, consistent with Cochrane handbook recommendations [27].

Heterogeneity in effect sizes among studies was checked via Cochrane’s I^2^ statistic. I² quantifies the proportion of total variation across studies due to true heterogeneity rather than sampling error. An I² value of 0% indicates no observed heterogeneity, whereas higher values (e.g., 25%, 50%, 75%) represent low, moderate, and high heterogeneity, respectively. Heterogeneity within our analysis ranged from 0 to 97.2%. Given the presence of high heterogeneity, we employed a random-effects model to account for between-study variance [32].

The results are presented as the SMD with a 95% confidence interval (CI). SMDs were classified as follows: between 0.20 and 0.49 as small, between 0.50 and 0.79 as moderate, and ≥ 0.8 as large [33].

## Results

### Article selection and characteristics

**Fig. 1:**
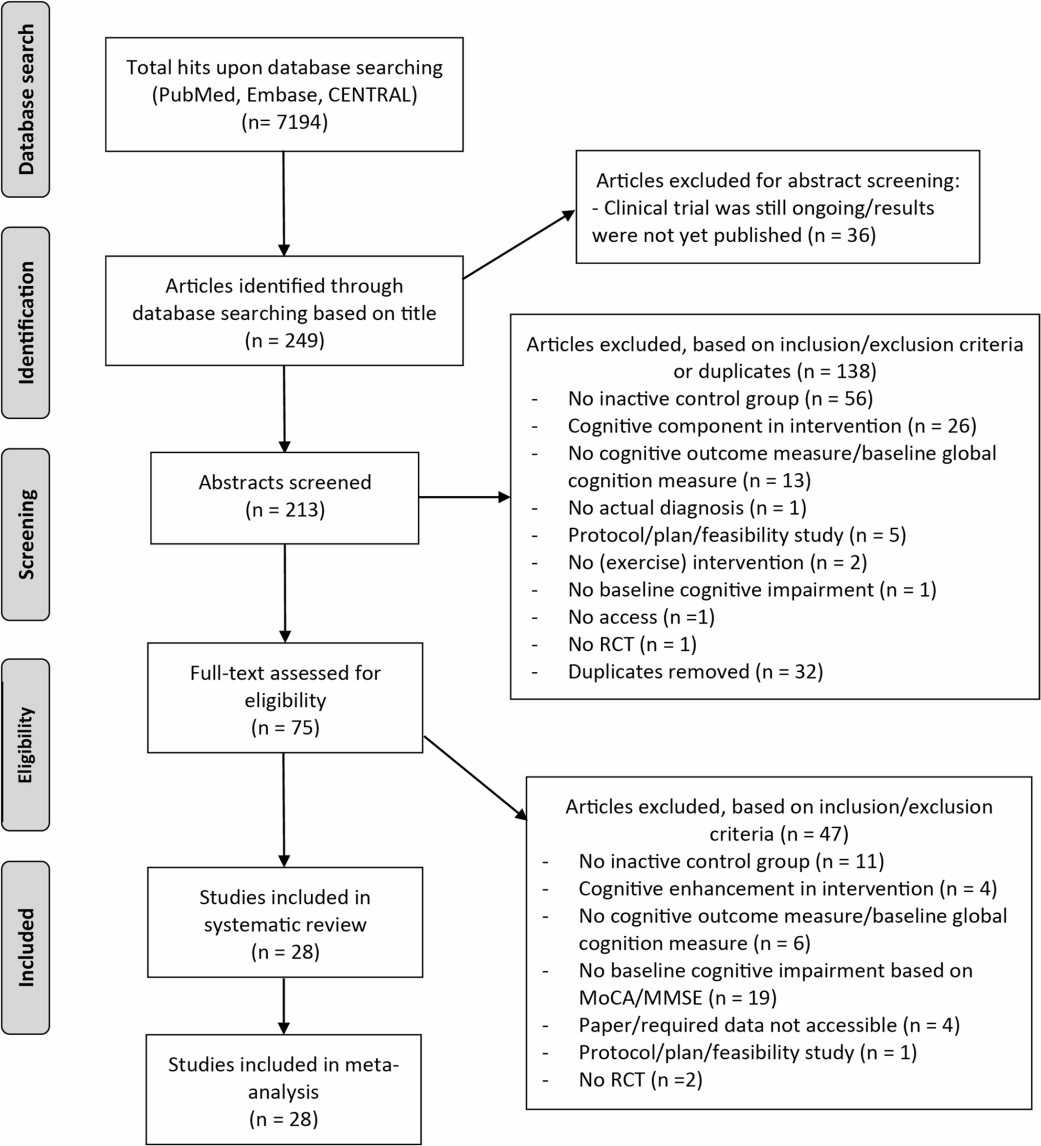
Flow chart of literature search and study selection

The database searches revealed 7194 primary hits. A total of 249 articles were deemed eligible based on their titles. After exclusion of RCTs that were still ongoing or did not yet publish any results, and screening of the remaining abstracts, 213 articles remained for full-text screening. This identified 28 articles that were considered eligible for this systematic review: eight articles included AD patients, 16 articles included MCI patients, and four articles included PD patients (Fig. 1).

The 28 eligible articles combined contained a total of 2088 subjects, of which 1133 subjects were assigned to an exercise intervention group and 955 subjects to a control group. The physical exercise modalities used in the studies included treadmill exercise, aerobic exercise, balance and strength training, dance exercise, cycling, resistance exercise, Tai Chi, walking, goal-based exercise and multicomponent exercise programs. The duration of these interventions ranged from three training sessions to 12 months of training sessions three times per week. All studies had cognition as a primary outcome measure. Global cognition was commonly assessed via the mini-mental state examination (MMSE) and the Montreal cognitive assessment (MoCA), whereas executive functions were commonly assessed via the Stroop test, verbal fluency test and the trail-making test B (TMT-B). Memory was often measured with the California verbal learning test (CVLT) and digit span forward, whereas attention tests mainly included the Stroop colour, Stroop word, or trail-making test A (TMT-A). Analyses were performed for each of these cognitive domains. An overview of the characteristics of all the studies included is shown in Table 1.

**Table 1:** Overview of study characteristics from all eligible studies

Paper	Subjects *N* per group (mean age in years)	Intervention/ frequency/ duration	Outcome measures:			
			Global cognition	Executive function	Memory	Attention
Arcoverde et al. 2014 [20]	AD patients IG = 10 (78.5) CG = 10 (79.0)	IG = 12 weeks treadmill exercise with progressive intensity and duration; 30 min/day, twice a week CG = No intervention	MMSE CAMCOG	Clock drawing test Verbal fluency category Stroop test Digit span backward	Digit span Digit span forward	TMT-A
de Oliveira Silva et al. 2019 [40]	AD patients IG = 13 (81.2) CG = 14 (77.5)	IG = 12 weeks of multimodal training sessions, comprised of balance, aerobic, and strength training + stretching; 60 min/day, twice a week CG = No intervention	MMSE	Verbal fluency test category	x	Stroop colour
Hoffmann et al. 2016 [23]	AD patients IG = 102 (69.8) CG = 88 (71.3)	IG = 16 weeks of 60 min moderate-to-high intensity aerobic exercise; 3 times/week CG = Usual treatment	MMSE ADAS-Cog (IR) ADAS-Cog (DR)	Stroop test incongruent Verbal fluency category Verbal fluency lexical	x	SDMT
Lok et al. 2023	AD patients IG = 36 (72.80) CG = 36 (73.88)	IG = 12 weeks of 30 min. musical exercise 3 days/week, 40 min. walking 2 days/week CG = No intervention	MMSE	x	x	x
Papatsimpas et al. 2023 [29]	AD patients IG1 = 57 (76.82) IG2 = 57 (76.07) CG = 57 (78.75)	IG1 = 12 weeks of aerobic (30 min/day, 5 days/week) and resistance (40–45 min/day, 3 days/week) exercise IG2 = 12 weeks of resistance exercise 40–45 min/day, 3 days/week CG = Usual activity	x	TMT-B Digit span backward	Digit span forward ACE-R memory	TMT-A ACE-R attention
Venturelli et al. 2011 [36]	AD patients IG = 116 (83) CG = 113 (85)	IG = 24 weeks of aerobic waking; 30 min/day, 4 days/week CG = No intervention	MMSE	x	x	x
Vreugdenhil et al. 2012 [39]	AD patients IG = 20 (73.5) CG = 20 (74.7)	IG = 16 weeks daily home-based exercises CG = Usual treatment	MMSE ADAS-Cog	x	x	x
Yang et al. 2015 [44]	AD patients IG = 25 (72.0) CG = 25 (71.9)	IG = 12 weeks cycling training; 40 min/day, 3 days/week CG = Usual treatment	MMSE ADAS-Cog	x	x	x
Bademli et al. 2019 [43]	MCI patients IG = 30 (72.7) CG = 30 (70.7)	IG = 20 weeks of a physical activity program warming up, rhythmic exercise, cooling down and walking CG = No intervention	MMSE	x	x	x
Chang et al. 2021 [48]	MCI patients IG = 62 (76.56) CG = 47 (75.94)	IG = 18 weeks of aerobic exercise; 30 min/day, 3 days/week CG = No intervention	MoCA	x	x	x
Hong, Kim, and Jun 2018 [46]	MCI patients IG = 10 (78.0) CG = 12 (76.7)	IG = 12 weeks of resistance exercise; 60 min/day, 2 days/week CG = No intervention	MoCA	COWAT – category fluency COWAT – letter fluency Stroop test incongruent Digit span backward	Digit span forward Rey 15-item recall Rey 15-item recognition	Stoop test congruent
Khanthong et al. 2021 [34]	MCI patients IG = 35 (60.26) CG = 36 (61.47)	IG = 12 weeks of traditional Thai exercise; 60 min/day, 3 days/week CG = No intervention	MoCA	Verbal fluency test TMT-B	x	TMT-A
Krootnark et al. 2024 [28]	MCI patients IG1 = 30 (68.60) IG2 = 30 (68.70) CG = 30 (69.70)	IG1 = 12 weeks of aerobic exercise 35 min/day, 5 days/week IG2 = 12 weeks of resistance exercise 35 min/day, 5 days/week CG = Usual activity	MoCA	TMT-B Stroop test incongruent Digit span backward	Digit span forward	TMT-A
Langoni et al. 2019 [45]	MCI patients IG = 26 (72.6) CG = 26 (71.9)	IG = 24 weeks of aerobic and strength exercises; 60 min per session CG = No intervention	MMSE	x	x	x
Li et al. 2021 [53]	MCI patients IG = 42 (60 +) CG = 42 (60 +)	IG = 6 months of multi-component exercise, 30 min/day, 5 days/week CG = Health education	MMSE MoCA	x	x	x
Li et al. 2022 [52]	MCI patients IG = 12 (73.93) CG = 12 (74.83)	IG = 8 week multicomponent exercise program; 60 min/day, 3 days/week CG = no intervention	ADAS-Cog	Colour trails test 2 Digit span backward	Digit span forward	Colour trails test 1
Liu et al. 2022 [54]	MCI patients IG1 = 17 (73.2) IG2 = 16 (74.6) CG = 17 (73.4)	IG1 = Tai Chi 12 weeks, 50 min/day, 3 days/week IG2 = Exergame Tai Chi weeks, 50 min/day, 3 days/week CG = Usual activity	MoCA	TMT-B TMT B-A Stroop test incongruent Stroop colour-word	CVLT short recall CVLT delayed recall	TMT-A
Lü et al. 2015 [35]	MCI patients IG = 22 (69.0) CG = 23 (70.4)	IG = 12 weeks of dumbbell training; 60 min/day, 3 days/week CG = Usual activity	ADAS-Cog	TMT-B Digit span backward	Digit span forward	x
Sánchez-Alcalá et al. 2025	MCI patients IG = 47 (71.43) CG = 45 (72.24)	IG = 12 week aerobic training program; 60 min/day, 2 days/week CG = Usual activity	MMSE MoCA	Verbal fluency category TMT-B	x	TMT-A
Song and Yu 2019 [42]	MCI patients IG = 60 (76.2) CG = 60 (75.3)	IG = 16 weeks of aerobic stepping exercise; 60 min/day, 3 days/week CG = Health education 2 times/week	MoCA	x	x	x
Stucken-schneider et al. 2021 [50]	MCI patients IG = 60 (70.6) CG = 58 (71.6)	IG = 12 months, aerobic exercise; 45 min/day, 3 days/week CG = No intervention	MoCA	TMT-B Letter fluency Category fluency	International shopping list International shopping list (delayed) One card learning task - Cogstate	TMT-A
Sungkarat et al. 2018 [47]	MCI patients IG = 29 (68.3) CG = 27 (67.5)	IG = 24 weeks, tai chi; 50 min/day, 3 days/week CG = No intervention	x	TMT-B-A Digit span forward/backward	Logical memory – delayed recall	x
Wang et al. 2020 [37]	MCI patients IG = 57 (68.37) CG = 54 (68.24)	IG = 12 weeks, structured limb-exercise program; 60 min/session, 3 days/week CG = Health promotion classes	MoCA	x	x	x
Yu et al. 2022 [30]	MCI patients IG1 = 10 (67.3) IG2 = 12 (67.2) CG = 12 (67.6)	IG1 = 24 weeks, Tai Chi, 60 min/session, 3/week IG2 = 24 weeks of aerobic and strengthening exercises 60 min/session, 3/week CG = Usual activity	HK-MoCA	TMT-B Verbal fluency category Stroop interference Digit span backward N-back task	Delayed recall test Digit span forward	TMT-A Stroop word Stroop colour
Avenali et al. 2019 [41]	PD patients IG = 17 (73.9) CG = 22 (71.2)	IG = 4 weeks, 6 × 60 min rehabilitation sessions/week CG = No intervention	MMSE MoCA	Weigl’s sorting test Frontal assessment battery TMT-B Phonological fluency Raven’s Matrices 1947	Verbal span Digit span CBTT	TMT-A
Harper et al. 2019 [51]	PD patients IG = 20 (65.1) CG = 15 (64.9)	IG = 3 times high cadence cycling for 40 min CG = No intervention	MoCA	Executive function (WebNeuro)	x	Attention/ concentration (WebNeuro)
Silveira et al. 2018 [31]	PD patients IG1 = 22 (70.63) IG2 = 21 (69.76) CG = 15 (67.60)	IG1 = 12 weeks aerobic exercise on cycle ergometers 60 min/day, 3 days/week IG2 = 12 weeks goal-based training, 60 min/day, 3 days/week CG = No intervention	x	TMT-B Digit span backward Stroop colour-word	Digit span forward Corsi block-tapping test CVLT short recall CVLT delayed recall Rey-O short recall Rey-O long recall	TMT-A Stroop word Stroop colour
Solla et al. 2019 [49]	PD patients IG = 10 (67.8) CG = 9 (67.1)	IG = 12 weeks dance program, 24 classes of 90 min. CG = Usual care	MoCA	x	x	x

*ACE-R* Addenbrooke’s cognitive examination, *ADAS-Cog* Alzheimer’s disease assessment scale-cognitive subscale, *AD* Alzheimer’s disease, *CAMCOG* Cambridge cognitive examination, *CBTT* Corsi block-tapping test, *CG* control group, *COWAT C*ontrolled oral word association test, *CVLT* California verbal learning test, *DR* delayed recall, *IG* intervention group, *IR* immediate recall, *MCI* Mild Cognitive Impairment, *MMSE* Mini Mental State Examination, *MoCA* Montreal Cognitive Assessment, *PD* Parkinson’s disease, *SDMT* symbol digit modalities test, *TMT* Trail-making test

### Participant characteristics

Of all participants (*N* = 2088) included in the studies, the mean ages of the patients ranged from 69.8 to 85 (AD), 65.1 to 73.9 (PD) and 60.3 to 78.8 (MCI) years (Table 1). Based on the average values of the MMSE and MoCA, all AD, PD and MCI patient groups presented with mild to moderate cognitive impairment before the onset of the intervention (Table 2). For all studies, patients with any condition that could preclude full participation in the physical exercise intervention, such as physical injuries, a disability or cardiovascular disease, were excluded. In addition, studies excluded participants who already engaged in physical activity regularly prior to the study. Seven studies reported several comorbidities in patients, such as hypertension, diabetes, hypercholesterolemia, stroke, and acute myocardial infarction [20, 23, 34–38], but these comorbidities were equally divided among the intervention and control groups. All other studies either excluded patients with any comorbidity that could have influenced the outcome [30, 31, 34, 39–42] or did not report on this [15, 28, 29, 43–53].

**Table 2:** Overview of baseline cognitive status of patient groups

Paper	Patient group	Cognitive test	Mean baseline score (+/- SD)
Arcoverde et al.	AD	MMSE	IG = 20.4 (2.7) CG = 19.9 (3.4)
De Oliveira Silva et al.	AD	MMSE	IG = 20.66 (5.19) CG = 20.90 (4.34)
Hoffmann et al.	AD	MMSE	IG = 23.8 (3.4) CG = 24.1 (3.8)
Lok et al.	AD	MMSE	IG = 23.36 (0.48) CG = 23.5 (0.5)
Papatsimpas et al.	AD	MMSE	IG1 = 20–24/30 IG2 = 20–24/30 CG = 20–24/30
Venturelli et al.	AD	MMSE	IG = 13 (2) CG = 12 (2)
Vreugdenhil et al.	AD	MMSE	IG = 22.9 (5.0) CG = 21.0 (6.3)
Yang et al.	AD	MMSE	IG = 21.33 (2.24) CG = 20 (3.5)
Bademli et al.	MCI	MMSE	IG = 23.27 (2.17) CG = 23.42 (1.07)
Chang et al.	MCI	MoCA	IG = 21.61 (2.11) CG = 21.49 (2.39)
Hong, Kim and Jun	MCI	MoCA	IG = 20.7 (3.46) CG = 20.08 (4.44)
Khanthong et al.	MCI	MoCA	IG = 20.31 (3.31) CG = 18.50 (2.92)
Krootnark et al.	MCI	MoCA	IG1 = 20.17 (2.09) IG2 = 19.60 (1.83) CG = 20.40 (1.87)
Langoni et al.	MCI	MMSE	IG = 21.9 (4.8) CG = 23.7 (3.7)
Li et al. (2021) [53]	MCI	MoCA	IG = 21.52 (2.05) CG = 21.14 (1.97)
Li et al. (2022) [52]	MCI	MoCA	IG = 22.61 (2.59) CG = 23.21 (2.40)
Liu et al.	MCI	MoCA	IG1 = 21.8 (3.6) IG2 = 22.6 (2.5) CG = 23.2 (2.8)
Lü et al.	MCI	MoCA	IG = 20.59 (2.92) CG = 20.96 (2.70)
Sánchez-Alcalá et al.	MCI	MoCA	IG = 21.36 (2.49) CG = 21.42 (1.03)
Song & Yu	MCI	MoCA	IG = 22.03 (1.81) CG = 22.10 (1.92)
Stuckenschneider et al.	MCI	MoCA	IG = 22.6 (2.5) CG = 22.4 (2.1)
Sungkarat et al.	MCI	MoCA	IG = 21.2 (3.4) CG = 20.4 (3.8)
Wang et al.	MCI	MoCA	IG = 21.65 (2.22) CG = 21.41 (2.11)
Yu et al.	MCI	MoCA	IG1 = 19.7 (1.5) IG2 = 19.3 (2.0) CG = 18.2 (3.8)
Avenali et al.	PD	MoCA	IG = 19.3 (0.88) CG = 17.43 (2.23)
Harper et al.	PD	MoCA	IG = 25.7 (2.8) CG = 25.7 (3.2)
Silveira et al.	PD	MoCA	IG1 = 25.22 (4.53) IG2 = 24.57 (3.99) CG = 25.80 (5.00)
Solla et al.	PD	MoCA	IG = 25.0 (3.97) CG = 25.67 (2.83)

*AD* Alzheimer’s disease, *CG* Control group, *IG* Intervention group, *MCI* Mild cognitive impairment, *MMSE* Mini mental state examination, *MoCA* Montreal cognitive assessment, *PD* Parkinson’s disease

Four studies excluded patients on medication that could influence cognition or any medication in general [31, 37, 47, 48]. Since avoiding medication use entirely was often not feasible, nine of the included studies permitted participants to continue their medication, provided that their usage was stable for at least a few months before and during the study [20, 23, 29, 36, 39, 41, 49–51]. In addition, medication use was reported to be consistent across both the intervention and control patient groups. Although stable medication use was also recommended by Silveira et al., some patients adjusted their medication, which was accounted for by including this as a covariate in their statistical analyses [31]. The remaining 14 studies did not provide information on medication use.

### Risk of bias in individual studies

**Fig. 2:**
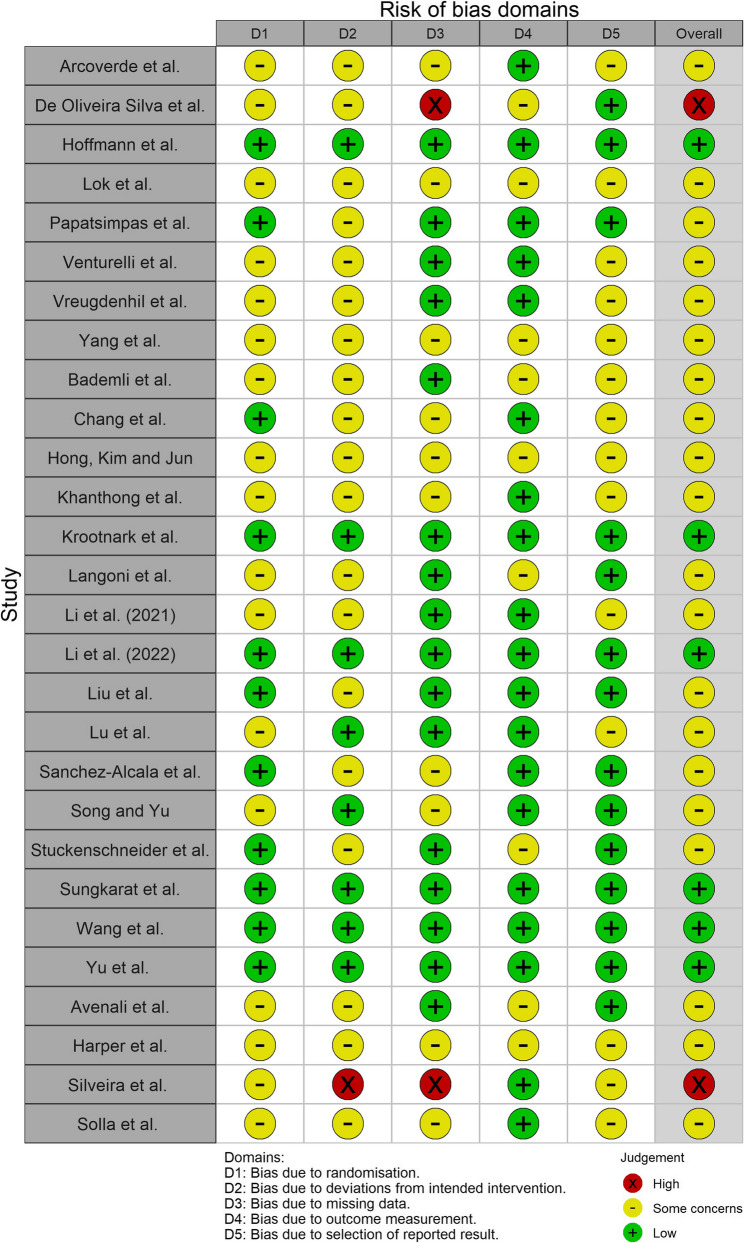
Risk of bias assessment plot The plot illustrates the risk of bias assessment for all RCTs included in the meta-analysis. Each row represents an individual study, and each column corresponds to a specific domain of bias evaluated, as specified in the legend. The colour-coded indicators reflect the level of risk for each domain: green for low risk of bias, yellow for some concerns and red for high risk of bias. The overall summary provides an aggregated view of the bias risk across all studies

Risk of bias in the individual studies is shown per domain (Fig. 2). Bias concerns were mainly due to a lack of a concealed allocation sequence, assessor blinding, intention-to-treat analysis, lack of outcome data or the lack of trial registration information. The studies by De Oliveira Silva et al. (AD) and Silveira et al. (PD) presented a high risk of bias in at least one of the domains [31, 40]. These studies were retained in the analysis, but their potential influence was explored in sensitivity analyses. Re-analysis excluding these high-risk studies did not alter the pooled effects for global cognition, executive function, or attention suggesting that the overall findings were robust. Removal of Silveira et al. did affect the outcome for memory in PD patients. However, this sub-domain analysis was based on only two studies in total, and should therefore be interpreted with caution. Notably, exclusion of the high-risk study resulted in a larger effect estimate, suggesting that its inclusion did not systematically inflate the observed effects. Among the other studies, six showed low risk of bias in all five domains [23, 28, 30, 37, 47, 53], and the remaining twenty studies showed some concerns in at least one of the domains.

### Effect of physical exercise on global cognition

**Fig. 3:**
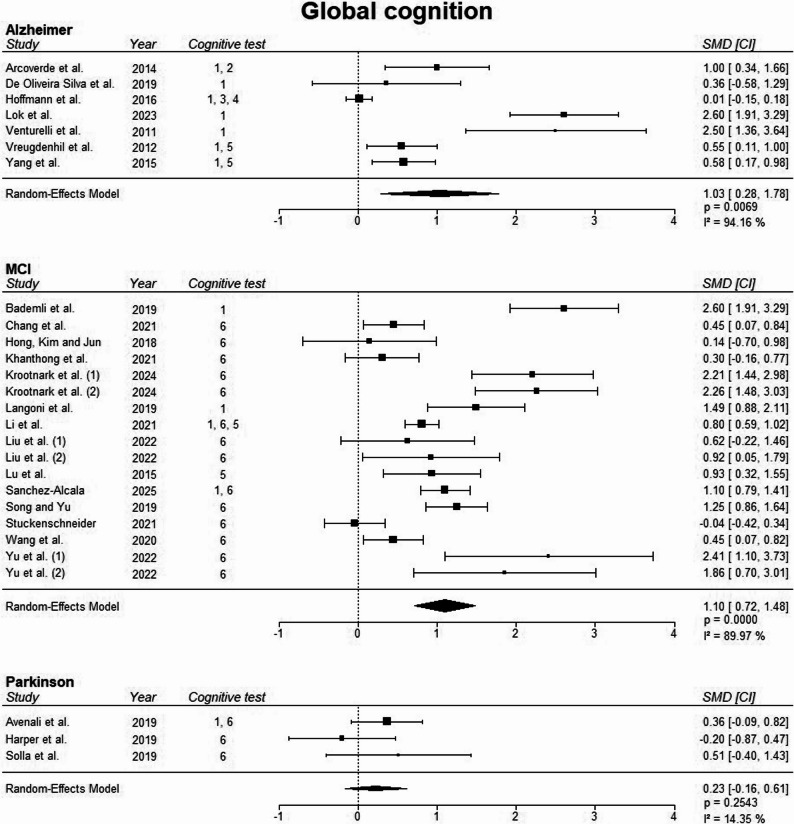
Effects of physical exercise interventions on global cognition in AD, PD and MCI patients This forest plot illustrates the effect sizes of physical exercise on global cognition across multiple studies. The x-axis represents the SMD, while the y-axis lists the individual studies included in the analysis. Each horizontal line represents the 95% confidence interval for the ES, and the filled black square indicates the point estimate of the ES. The size of each square corresponds to the weight of the study in the meta-analysis. The black diamond at the bottom of the plot represents the pooled ES with its 95% confidence interval. The vertical black line denotes the null effect. Studies to the right of this line favour the exercise intervention, whereas those to the left favour no intervention. Cognitive tests are represented by a number: 1 = MMSE, 2 = CAMCOG, 3 = ADAS-Cog (IR), 4 = ADAS-Cog (DR), 5 = ADAS-Cog, 6 = MoCA. The different intervention groups of studies that included multiple exercise interventions are indicated with (1) or (2), and correspond to IG1 or IG2, respectively, as presented in Table 1

Twenty-four studies examined the effects of exercise on global cognition in patients with AD, MCI or PD. In total, 36 cognitive tests were performed, comprising the Alzheimer’s disease assessment scale–cognitive subscale (ADAS-Cog), Cambridge cognitive examination (CAMCOG), MMSE and MoCA. As shown in Fig. 3, a small, non-significant effect of physical exercise on global cognition was found in PD patients (*N* = 93, SMD = 0.23, 95% CI -0.16–0.61, *p* = 0.2543). In contrast, a significant, large positive effect of physical exercise was observed among AD patients (*N* = 628, SMD = 1.03, 95% CI 0.28–1.78, *p* = 0.0069) andMCI patients (*N* = 1082, SMD = 1.13, 95% CI 0.73–1.53, *p* < 0.0001).

### Effect of physical exercise on executive function

**Fig. 4:**
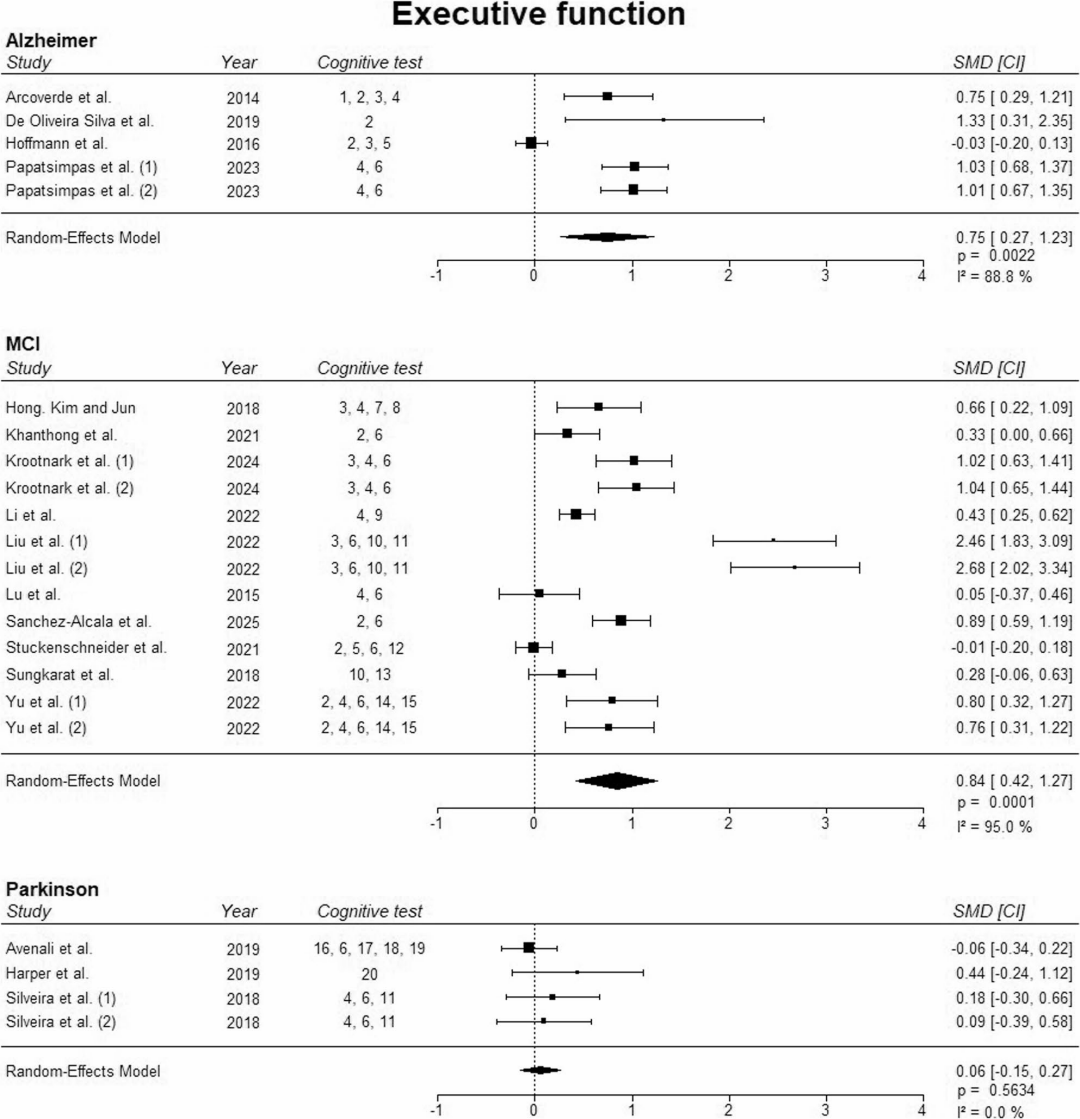
Effects of physical exercise interventions on executive function in AD, PD and MCI patients This forest plot illustrates the effect sizes of physical exercise on executive function across multiple studies. The x-axis represents the SMD, while the y-axis lists the individual studies included in the analysis. Each horizontal line represents the 95% confidence interval for the ES, and the filled black square indicates the point estimate of the ES. The size of each square corresponds to the weight of the study in the meta-analysis. The black diamond at the bottom of the plot represents the pooled ES with its 95% confidence interval. The vertical black line denotes the null effect. Studies to the right of this line favour the exercise intervention, whereas those to the left favour no intervention. Cognitive tests are represented by a number: 1 = clock drawing test, 2 = verbal fluency-category, 3 = Stroop test incongruent, 4 = digit span backward, 5 = verbal fluency-lexical, 6 = TMT-B, 7 = COWAT-semantic fluency, 8 = COWAT-phonemic fluency, 9 = colour trails test 2, 10 = TMT B-A, 11 = Stroop colour-word, 12 = one back task – Cogstate, 13 = digit span forward/backward, 14 = Stroop interference, 15 = n-back task, 16 = frontal assessment battery, 17 = Weigl’s sorting test, 18 = phonological fluency, 19 = Raven’s matrices 1947, 20 = executive function (WebNeuro). The different intervention groups of studies that included multiple exercise interventions are indicated with (1) or (2), and correspond to IG1 or IG2, respectively, as presented in Table 1

Seventeen studies examined the effects of physical exercise on executive function in AD, MCI or PD patients. In total, 69 cognitive tests were performed including the verbal fluency test, TMT-B, and Stroop colour-word test. No effect of exercise was found in PD patients (*N* = 132, SMD = 0.06, 95% CI -0.15–0.27, *p* = 0.5634). A significant, moderate effect of physical exercise on executive function was found in AD patients (*N* = 408, SMD = 0.75, 95% CI 0.27–1.23, *p* = 0.0022). A significant, large positive effect of exercise was found in MCI patients (*N* = 602, SMD = 0.84, 95% CI 0.42–1.27, *p* < 0.0001) (Fig. 4).

### Effect of physical exercise on memory

**Fig. 5:**
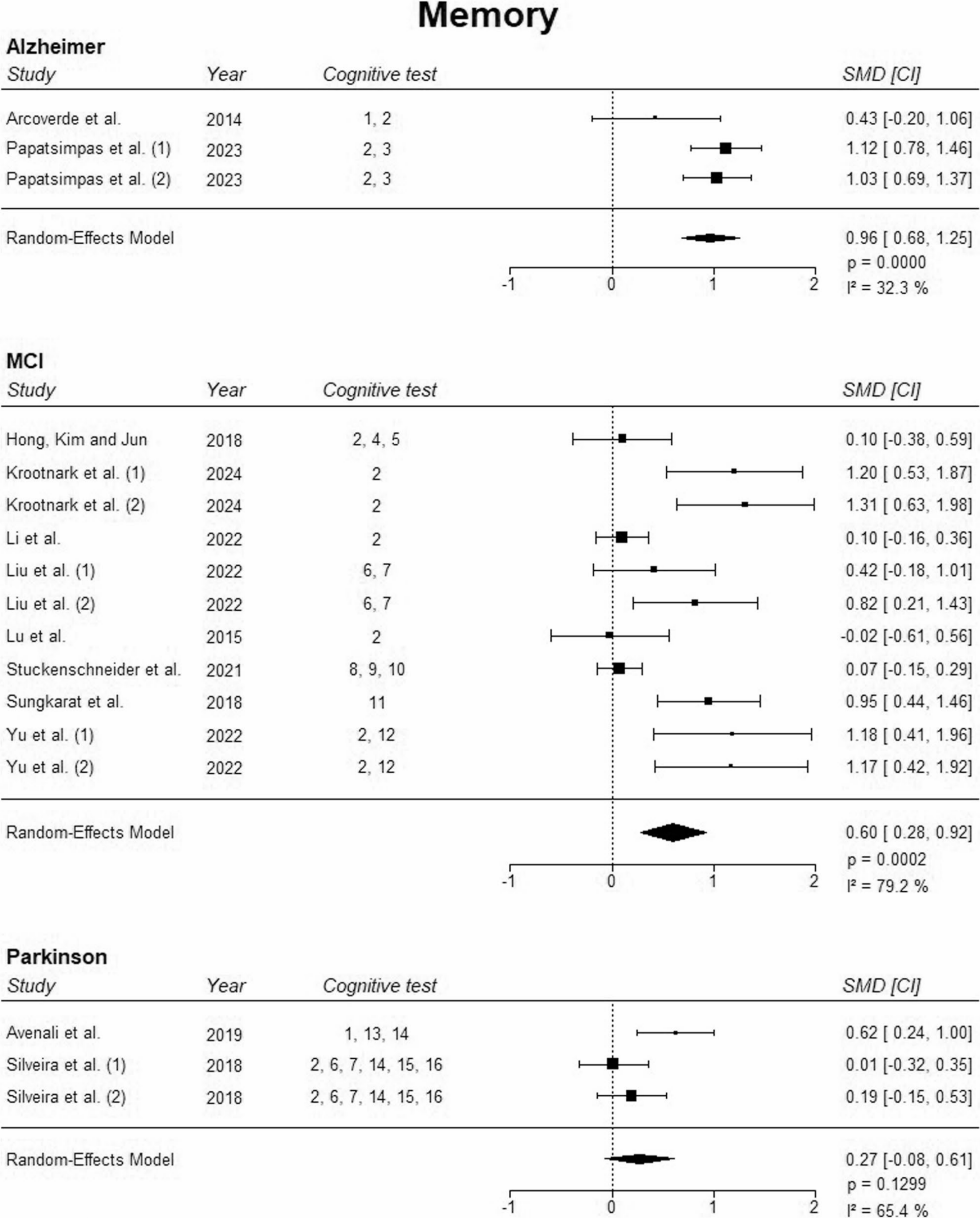
Effects of physical exercise interventions on memory in AD, PD and MCI patients This forest plot illustrates the effect sizes of physical exercise on memory across multiple studies. The x-axis represents the SMD, while the y-axis lists the individual studies included in the analysis. Each horizontal line represents the 95% confidence interval for the ES, and the filled black square indicates the point estimate of the ES. The size of each square corresponds to the weight of the study in the meta-analysis. The black diamond at the bottom of the plot represents the pooled ES with its 95% confidence interval. The vertical black line denotes the null effect. Studies to the right of this line favour the exercise intervention, whereas those to the left favour no intervention. Cognitive tests are represented by a number: 1 = digit span, 2 = digit span forward, 3 = ACE_R memory, 4 = Rey 15-item test (short term memory), 5 = Rey 15-item test (recognition memory), 6 = CVLT short recall, 7 = CVLT delayed recall, 8 = verbal memory (international shopping list Cogstate), 9 = verbal memory (international shopping list delayed Cogstate), 10 = one card learning task – Cogstate, 11 = logical memory – delayed recall, 12 = delayed recall test, 13 = verbal span, 14 = CBTT, 15 = Rey-O short recall, 16 = Rey-O long recall. The different intervention groups of studies that included multiple exercise interventions are indicated with (1) or (2), and correspond to IG1 or IG2, respectively, as presented in Table 1

Twelve studies examined the effects of physical exercise on memory in AD, MCI or PD patients. In total, 40 cognitive tests were performed including the digit span forward, Corsi block tapping test (CBTT). A significant, large positive effect was observed in AD patients (*N* = 191, SMD = 0.96, 95% CI 0.68–1.25, *p* < 0.0001). Exercise led to a significant, moderate positive effect on memory in MCI patients (*N* = 439, SMD = 0.60, 95% CI 0.28–0.92, *p* = 0.0002). A small, non-significant positive effect was seen in PD patients (*N* = 97, SMD = 0.27, 95% CI -0.08–0.61, *p* = 0.1299) (Fig. 5).

### Effect of physical exercise on attention

**Fig. 6:**
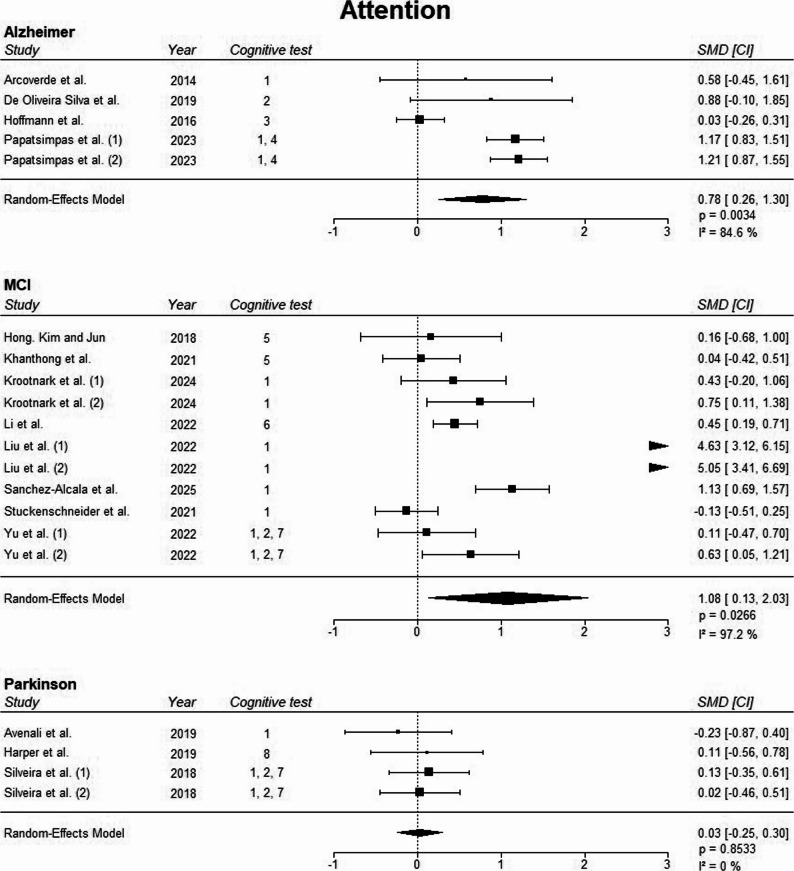
Effects of physical exercise interventions on attention in AD, PD and MCI patients This forest plot illustrates the effect sizes of physical exercise on attention across multiple studies. The x-axis represents the SMD, while the y-axis lists the individual studies included in the analysis. Each horizontal line represents the 95% confidence interval for the ES, and the filled black square indicates the point estimate of the ES. The size of each square corresponds to the weight of the study in the meta-analysis. The black diamond at the bottom of the plot represents the pooled ES with its 95% confidence interval. The vertical black line denotes the null effect. Studies to the right of this line favour the exercise intervention, whereas those to the left favour no intervention. Cognitive tests are represented by a number: 1 = TMT-A, 2 = Stroop colour, 3 = SDMT, 4 = ACE-R attention and orientation, 5 = Stroop test congruent, 6 = colour trails test 1, 7 = Stroop word, 8 = attention/concentration (WebNeuro). The different intervention groups of studies that included multiple exercise interventions are indicated with (1) or (2), and correspond to IG1 or IG2, respectively, as presented in Table 1

Fifteen studies examined the effects of physical exercise on attention in AD, MCI and PD patients. In total 30 cognitive tests were performed, comprising mainly of the TMT-A, Stroop word, and Stroop colour. No effect of physical exercise was observed in PD patients (*N* = 132, SMD = 0.03, 95% CI -0.25–0.30, *p* = 0.8533). Exercise interventions led to a significant, moderate positive effect in AD patients (*N* = 381, SMD = 0.78, 95% CI 0.26–1.30, *p* = 0.0034). A significant, large positive effect was observed in MCI patients (*N* = 501, SMD = 1.08, 95% CI 0.13–2.03, *p* = 0.0266) (Fig. 6).

## Discussion

This meta-analysis examined the effects of physical exercise on cognitive functioning in patients with Alzheimer’s disease (AD), Parkinson’s disease (PD) or mild cognitive impairment (MCI) who already showed objective cognitive impairment before the start of the intervention. Because these conditions differ in cognitive profile and disease course, we analysed four cognitive domains that are relevant across all groups: global cognition, executive function, memory and attention. Overall, our findings demonstrate that physical exercise is associated with improvements in cognition across multiple domains. Significant improvements were observed in global cognition, executive function, memory and attention in both AD and MCI patients, whereas no significant effects were found in PD patients, although effect estimates were generally in a positive direction. Notably, these beneficial effects were observed despite substantial variability in exercise interventions across studies, suggesting that cognitive benefits may extend across different types of exercise. These findings support the potential of physical exercise as a strategy to improve or maintain cognitive functioning in neurodegenerative conditions.

In AD, physical exercise was associated with significant improvements across all assessed cognitive domains, including global cognition, executive function, memory and attention, in line with previous meta-analyses [54, 55]. These findings suggest that exercise-related benefits are not limited to general cognitive functioning but may extend to more specific cognitive processes. However, the number of studies contributing to some domain-specific analyses was relatively limited, and variability in cognitive outcome measures across studies may have influenced the observed effects. This is partly explained by the fact that many AD studies primarily assessed global cognition, resulting in a larger number of studies contributing to this domain compared to more specific cognitive outcomes. As a result, although improvements were consistently observed, the precision of estimates for domain-specific outcomes remains limited due to the smaller number of contributing studies.

In MCI, physical exercise was also associated with significant improvements across all analysed cognitive domains. The relatively large number of included studies in this group likely contributed to more precise and stable effect estimates. In addition, findings were generally consistent across studies, supporting the robustness of the observed effects.

In PD, no significant effects were observed across the analysed domains. This contrasts with previous meta-analyses reporting beneficial effects of exercise on cognition in PD [56], which often included combined physical and cognitive interventions. In the present study, only exercise-only interventions were considered, and only four studies met the inclusion criteria. This limited number of studies substantially reduced statistical power and restricts the ability to draw firm conclusions. Therefore, the findings in PD should be interpreted as inconclusive rather than as evidence of absence of effect.

An important observation from this meta-analysis is the limited number of randomized controlled trials that specifically include patients with objectively verified cognitive impairment at baseline. Although this criterion increased the clinical relevance of the present study, it substantially reduced the number of eligible studies, particularly in PD. In AD, the number of studies contributing to domain-specific analyses was further limited by the tendency of many trials to focus primarily on global cognition, resulting in fewer studies reporting outcomes for specific cognitive domains.

Overall, the present findings support the view that physical exercise is a promising and accessible non-pharmacological strategy for supporting cognitive function in individuals with neurodegenerative disorders who already exhibit cognitive impairment. The consistency of findings across multiple cognitive domains, despite variability in exercise interventions, strengthens the case for incorporating exercise into clinical practice. At the same time, the limited availability of studies including patients with objectively verified cognitive impairment, as well as the focus on global cognition in many AD studies, highlights the need for further high-quality research to refine effect estimates and better characterize domain-specific and disease-specific responses.

### Limitations and recommendations

Several limitations should be considered when interpreting the findings of this meta-analysis. First, we selected RCTs in which patient groups showed objective cognitive impairment before the exercise intervention based on reported mean baseline cognitive scores. Although this approach allowed us to focus on clinically relevant populations with cognitive complaints, classification at the group level has limitations. In some studies, the standard deviations around MMSE or MoCA scores indicate that not all individual participants were necessarily cognitively impaired according to the predefined thresholds. Moreover, MMSE and MoCA are broad screening tools and may not capture impairment equally well across different neurodegenerative disorders. As a result, some degree of heterogeneity in baseline cognitive status likely remained.

Second, there was considerable variation in exercise type, duration, intensity, training frequency, and cognitive outcome measures across studies. This clinical and methodological heterogeneity may have diluted true effects, contributed to the wide range in observed heterogeneity, and limited the ability to determine which exercise regimens are most effective for specific patient groups or cognitive domains. Future research would benefit from more standardized exercise protocols and harmonized, domain-specific cognitive test batteries.

Third, a methodological consideration concerns the handling of multiple cognitive outcomes within individual studies. Although hierarchical (multilevel) meta-analysis is generally the preferred approach for accounting for dependency among multiple effect sizes from the same study, this was not feasible in the present analysis. Such models require either within-study correlations between outcomes or sufficient numbers of studies and effect sizes per domain to estimate variance components reliably. In our dataset, within-study correlations were not reported and could not be estimated with confidence, and the number of studies in several subgroup analyses was limited. We therefore combined outcomes within domains using a weighted average of standardized mean differences (SMDs), a pragmatic and commonly used approach when dependency information is unavailable [27]. This method prevents studies with multiple outcomes from being overrepresented, but it does not fully account for within-study dependence. Consequently, the variance of pooled estimates may have been underestimated, which could result in narrower confidence intervals and a higher risk of Type I error. The precision of the pooled effects should therefore be interpreted cautiously.

Finally, the number of eligible studies was unevenly distributed across disease groups and domains, with especially few studies available for PD. This limits statistical power and makes the absence of significant findings difficult to interpret. In addition, because the present analyses were conducted separately by disease group without formal interaction testing, the results should not be used to draw direct conclusions about comparative responsiveness to exercise between AD, PD, and MCI.

## Conclusion

In this meta-analysis, physical exercise was associated with significant improvements in global cognition, executive function, memory and attention in patients with AD and MCI who already exhibited cognitive impairment at baseline. In PD patients, no significant cognitive improvements were observed, although effect estimates were generally in a positive direction, likely reflecting the limited number of available studies.

Notably, these beneficial effects were observed despite substantial variability in exercise interventions across studies, suggesting that exercise may have broadly applicable cognitive benefits across different modalities. These findings support physical exercise as a promising and accessible non-pharmacological strategy for individuals with neurodegenerative disorders.

At the same time, the limited number of studies including patients with objectively verified cognitive impairment, particularly in PD, and the predominant focus on global cognition in many AD studies, highlight important gaps in the current literature. Future randomized controlled trials should build on these findings by specifically targeting cognitively impaired populations and including sufficiently large and well-characterized samples to improve the precision and clinical applicability of the evidence.

## Data Availability

The data used and/or analysed in the study are available from the corresponding author on reasonable request.

## Abbreviations

ACE-R: Addenbrooke’s cognitive examination
AD: Alzheimer’s disease
ADAS-Cog: Alzheimer’s disease assessment scale-cognitive subscale
BDNF: Brain-derived neurotrophic factor
CAMCOG: Cambridge cognitive examination
CBTT: Corsi block-tapping test
CENTRAL: Cochrane Central Register of Controlled Trials
CG: Control group
CI: Confidence interval
COWAT: Controlled oral word association test
CVLT: California verbal learning test
DR: Delayed recall
ES: Effect size
FAB: Frontal assessment battery
IG: Intervention group
IGF-1: Insulin-like growth factor 1
IR: Immediate recall
MCI: Mild cognitive impairment
MMSE: Mini-mental state examination
MoCA: Montreal cognitive assessment
PD: Parkinson’s disease
RCT: Randomized controlled trial
RoB: Risk of bias
SD: Standard deviation
SDMT: Symbol digit modalities test
SMD: Standardized mean difference
TMT: Trail-making test
VEGF: Vascular endothelial growth factor
